# Geographic Patterns of Inversion Polymorphism in the Second Chromosome of the Cactophilic *Drosophila buzzatii* from Northeastern Argentina

**DOI:** 10.1673/031.010.14141

**Published:** 2010-10-20

**Authors:** Ignacio M. Soto, Eduardo M. Soto, Valeria P. Carreira, Juan Hurtado, Juan J. Fanara, Esteban Hasson

**Affiliations:** Departamento de Ecología, Genética y Evolución. Facultad de Ciencias Exactas y Naturales. Universidad de Buenos Aires. Argentina

**Keywords:** cactus, clines, geographic variation, natural selection, *Opuntia*

## Abstract

The inversion polymorphisms of the cactophilic *Drosophila buzzatti* Patterson and Wheeler (Diptera: Drosophilidae) were studied in new areas of its distribution in Argentina. A total of thirty-eight natural populations, including 29 from previous studies, were analyzed using multiple regression analyses. The results showed that about 23% of total variation was accounted for by a multiple regression model in which only altitude contributed significantly to population variation, despite the fact that latitude and longitude were also included in the model. Also, inversion frequencies exhibited significant associations with mean annual temperature, precipitation, and atmospheric pressure. In addition, expected heterozygosity exhibited a negative association with temperature and precipitation and a positive association with atmospheric pressure. The close similarity of the patterns detected in this larger dataset to previous reports is an indication of the stability of the clines. Also, the concurrence of the clines detected in Argentina with those reported for colonizing populations of Australia suggests the involvement of natural selection as the main mechanism shaping inversion frequencies in *D. buzzatii*.

## Introduction

The study of inversion polymorphisms in the genus *Drosophila* has long constituted a model system to study the adaptive processes involved in the maintenance of genetic variation. The pioneering work of Dobzhansky and colleagues with inversions in *D. pseudoobscura* and allied species (see Dobzhansky 1970) was influential in the construction of the Modern Synthesis. Since then, stable inversion polymorphisms have been detected in a large number of insects (Hoffman et al. 2004) and frequently used as model systems for the study of natural selection in the wild and of associations between inversions and fitness-related traits (Dobzhansky 1970; Lewontin et al. 1981; Krimbas and Powell 1992; Hoffman et al. 2004; Ayala and Coluzzi 2005).

One of these model systems is *D. buzzatii* Patterson and Wheeler (Diptera: Drosophilidae), a cactophilic species that breeds and feeds in the necroses of Cactaceae (Barker 1977; Barker and Starmer 1982; Fontdevila et al. 1982; Barker et al. 1985; Hasson et al. 1995). Patterns of variation of inversion frequencies were investigated in both the original area of the species range (Fontdevila et al. 1982; Hasson et al. 1995) and in colonizing populations of the Old World (Fontdevila et al. 1981) and Australia (Knibb et al. 1987). Thirteen gene orders have been described for *D. buzzatii*'s second chromosome. Of those *2st, 2j*, and *2jz^3^* are the most common and widely distributed, while other arrangements such as *2jq^7^* and *2y*^3^ are restricted to very few locations in Argentina (Hasson et al. 1995).

Various studies have explored the relationship between second chromosome inversions and body size, developmental time, viability, and longevity (Rodriguez et al. 1999; Fernández Iriarte and Hasson 2000). These studies showed that *2st*, the ancestral arrangement, decreases body size and accelerates egg to adult development whereas derived arrangements of the *2j* phylad affects the same traits, but in the opposite direction. Further studies have found that associations of inversion arrangements with quantitative traits (viability, developmental time, and body size) depend on the host cactus (Fernández Iriarte and Hasson 2000; Fernández Iriarte et al. 2003). Moreover, artificial selection for fast developing flies coupled with selection for small or large body size revealed correlated responses of the inversion polymorphism in the expected directions. On one hand, the frequency of *2st* increased in lines selected for rapid development and small body size, but was lost when selection operated in the same direction for developmental time in the opposite body size (Cortese et al. 2002; unpublished results).

Furthermore, *D. buzzatii* has proved to be a fruitful model for the study of natural selection in the wild since the knowledge of its breeding sites allows the assessment of changes of inversion frequencies during the life cycle. This kind of study has shown that inversions affect several fitness components and that some of these effects are population specific (Ruiz et al. 1986; Hasson et al. 1991). Moreover, it has been proposed that antagonistic pleiotropy may be involved in the maintenance of the inversion polymorphism since the effects of each gene arrangement on different fitness components are negatively correlated (Ruiz et al. 1986; Hasson et al. 1991). For instance, *2jz^3^* increases longevity and impairs fecundity; while *2st* confers greater pupal viability and decreases adult viability (Hasson et al. 1991; Rodriguez et al. 1999).

Surveys of inversion frequencies in natural populations of *D. buzzatii* revealed latitudinal and altitudinal clines, and population differentiation consistent with major phytogeographic divisions in Argentina (Hasson et al. 1995).

All this evidence suggests that natural selection may be the major factor contributing to population structure. However, it should be noted that studies performed in the last two decades focused mainly on collections performed in the arid regions of northwestern Argentina, an area that includes subtropical to warm temperate desertic and semidesertic environments which are collectively known as the Monte biome (Fernández and Busso 1997). This region extends from 24° 35′ S to 44° 20′ S and is limited by the Andes in the west, the Patagonian semidesertic area in the south, and the dry subtropical woodlands of Chaco and Espinal in the east (Cabrera 1976).

In this study, the results of new collections in the semiarid, more humid northeastern region extend the sampling area to more central locations of the Monte. With this extended dataset the stability of clines was tested. This reexamination of patterns of variation of inversion frequencies confirms the stability of altitudinal clines and broadens knowledge of biogeographic patterns of variation in inversion frequencies.

## Materials and Methods

In this paper, second chromosome inversion frequencies were obtained from 10 natural populations of *D. buzzatii* sampled in late Summer 2007 (Table 1). *D. buzatti* were collected by net sweeping over yeasted-banana baits. Collected females were transported to the laboratory and placed in individual vials (isofemale lines) containing a modified formula of David's killed yeast medium (David 1962). Isofemale lines were maintained under controlled conditions of temperature (25 ± 1° C) and photoperiod (12:12 L:D). Species were identified by E. Hasson and FM Soto by examining the genitalia of one male progeny of each isofemale line since *D. buzzatii* females cannot be morphologically distinguished from females of the closely related *D. koepferae* (Vilela 1983).

Cytological characterization of each isofemale line was accomplished via the analysis of the polytene chromosomes of one progeny larva (Heed and Carson 1983). Salivary gland chromosome preparations were obtained according to Fontdevila et al. (1981) and observed in a light microscope at 400× magnification.

Patterns of variation of inversion frequencies were investigated by means of multiple regression analysis using the package Statistica (Statsoft Inc. 1995). The associations were tested between the three most common second chromosome arrangements (2standard-*st*-, *2j*, and *2jz* ^3^) and expected heterozygosis (*H*) [dependent variables] with geographic or climatic variables [independent variables]. Geographical variables considered were latitude, longitude, and altitude (obtained ‘in situ’ using a GPS) and climatic variables were mean annual temperature, mean annual precipitation (mm/day), and mean annual atmospheric pressure averaged over the last 10 years (or the nearest available period) obtained from the Royal Netherlands Meteorological Institute public web site (KNMI, http://www.knmi.nl/). Regression analyses were performed using the package Statistica (Statsoft Inc. 1995). Arrangement frequencies and expected heterozygosity were angulary transformed prior to statistical analysis.

**Table 1.**
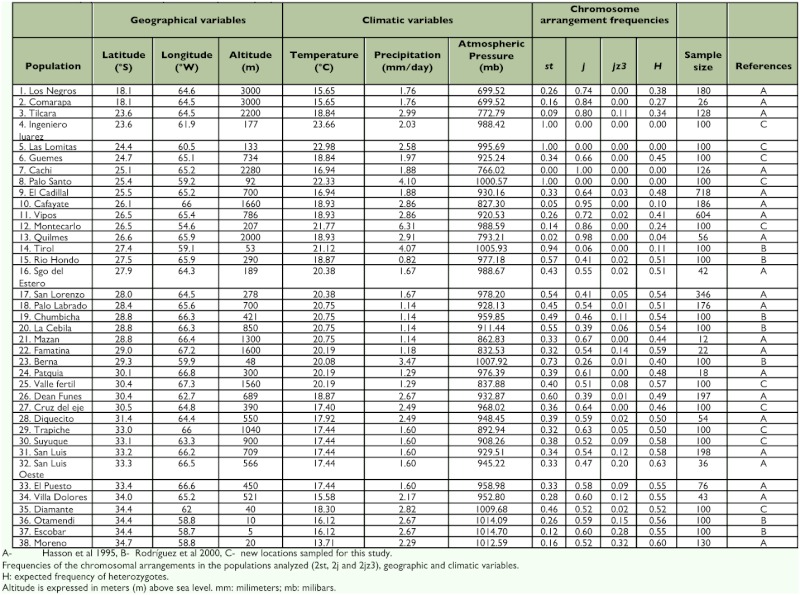
Natural populations of *Drosophila buzzatii* previously reported.

## Results

**Figure 1.**
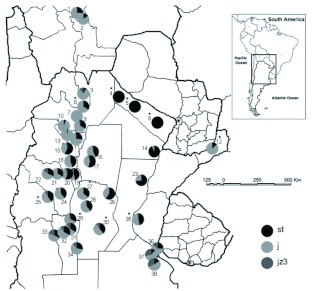
Collection sites and inversion frequencies (depicted as percentages) in natural populations of *Drosophila buzzatii. st* is standard, *j* and *jz^3^* are the arrangements detected in the survey. 1–38: Location reference number (see Table 1). Stars indicate newly collected populations. High quality figures are available online.

The frequencies of the three most common arrangements varied among sampling localities (Table 1). The ancestral gene arrangement, designated as *2st*, was most abundant in northeastern localities (labelled as 4, 5, 8, 14, and 23) reaching its maximum frequency in Ingeniero Juarez, Palo Santo and Las Lomitas (localities 4, 5, and 8 in Figure 1), where it is apparently fixed. The only exception to this trend among northeastern localities was Montecarlo (#12), where the most abundant gene order was the derived arrangement *2j*. However, the population inhabiting this area probably represents a recent colonization since flies live upon the necrotic cladodes of the introduced prickly pear *Opuntia ficus-indica* (JJ Fanara personal communication). Inversion frequencies in Valle Fértil (site #25), Cruz del Eje (#27), Trapiche (#29), and Suyuque (#30) were very similar, *2j* (reaching values that were greater than 0,5) and *2st* (reaching values that were not lower than 0.32) were the most common arrangements in these sampling sites, while *2jz^3^* was rare. Considering the entire picture of all populations sampled so far, the inversion polymorphism in Central Chaco mainly involves two arrangements in intermediate frequencies (localities 15–19 and 26). Moving away from this region, the frequency of *2j* increases steadily towards the Northwest reaching its maximum in Cachi (# 7, where it is fixed), Cafayate and Quilmes (#10 and #13) where it is almost fixed, and decreases both towards the North East where it is almost absent and the South. On the contrary *2st* exhibits the complementary trend. Finally, *2jz^3^* was absent in the Northeast and rare in the Northwest, and increased towards the Southwest and Southeast borders of the species range in Argentina (Table 1, Figure 1).

First, patterns of variation in inversion frequencies were examined by means of multiple regression analyses of inversion frequencies as dependent variables on geographic and climatic variables for the set of newly sampled localities. Even though the biogeographic trends detected were concordant with the results reported in previous studies based on larger sets of populations (Hasson et al. 1995), regression analysis did not reveal significant results (Data not shown). This was probably due to the small number of localities analyzed. Thus, the researchers decided to perform multiple regression analyses including newly sampled localities along with the dataset used by Hasson et al. (1995). Thus, regarding geographic variables, about 23% (averaged across arrangements) of total population variation could be accounted for by a multiple regression model including latitude, longitude, and altitude. However, the significance of the regression model could be mainly accounted for by altitude. In effect, the frequency of *2st* was negatively correlated with altitude whereas *2j* exhibited the opposite trend (in both cases the partial regression accounted for 25% of total variance) (Table 2a).

Multiple regression analysis of inversion frequencies on climatic variables revealed even stronger associations accounting for more than 48% (averaged across arrangements) of total among population variance. All climatic variables contributed significantly to the regression model (Table 2b). *2st* was positively correlated with both mean annual temperature and atmospheric pressure, and *2j* exhibited the opposite trends. Finally, *2jz*^3^ was negatively associated with temperature and positively correlated with atmospheric pressure. Furthermore, expected heterozygosity, which was only weakly associated with longitude (Table 2a), was significantly correlated with climatic variables (Table 2b). Heterozygosity was negatively associated with temperature and precipitation, and positively associated with atmospheric pressure.

However, temperature, precipitation, and atmospheric pressure are undoubtedly associated with altitude. Thus, a multifactorial regression analysis was done including both geographic and climatic variables, since this approach allows testing of individual factors after adjusting for all other terms in the model. This analysis showed that about 66% (averaged across arrangements) of total population variation could be accounted for by a model that included latitude, longitude, altitude, temperature, precipitation, and atmospheric pressure. However, a close examination of partial regressions revealed different patterns for the three arrangements. On one hand, patterns of variation *2st* and *2j* were mainly correlated with climatic variables, especially temperature and precipitation. *2st* tends to be more frequent in warmer locations, while *2j* increases its frequency in cooler and drier environments. On the other hand, the main determinants of variation of *2jz^3^* were latitude, longitude, and precipitation. In effect, this arrangement was more frequent in eastern and southern areas which also happen to be more humid in the wide area that inhabits *D. buzzatii* in Argentina and Bolivia.

## Discussion

Clinal variation along geographical and/or climatic gradients of both phenotypic and genetic variants are often accepted as partial evidence of selective differentiation. However, parallel or reciprocating clines (opposing clines in northern and southern hemispheres) in different areas of a widely distributed species are assumed to be strong arguments in favor of differential selection along environment gradients.

**Table 2.**
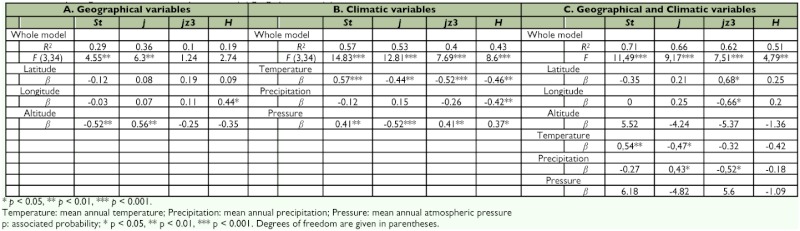
Multiple regression of inversion frequencies on (A) geographical and (B) climatic variables.

Parallel patterns of geographical variation in inversion frequencies in different continents along geographical or climatic gradients have been documented in many species of *Drosophila* and other insects (Krimbas and Powell 1992; Powell 1997; Hoffmann et al. 2004). The palearctic *D. subobscura* is perhaps the most spectacular example in this matter, since inversions frequencies in the newly colonized areas of South and North America converged very rapidly to the latitudinal clines described in Europe and Northern Africa, the original area of the species. Moreover, the latitudinal clines and the seasonal changes in inversion frequencies reported in *D. subobscura* have been proposed as an indicator of global warming. Actually, a time series analysis showed that changes in inversion frequencies in the last 35 years in Europe could be due to climatic factors that made the chromosomal composition of this species more ‘southern’ (Solé et al. 2002). This gives support to the proposal that inversion polymorphisms may be considered as early warning systems for climate changes well before populations are threatened (reviewed in Hoffmann et al. 2004).

Previous surveys of the inversion polymorphism in *D. buzzatii* comprised a large number of natural populations located predominantly in the arid northwestern portion of the species' range (Hasson et al. 1995). Here the study was extended by including new sampling areas underrepresented in previous studies like the northeastern and midwestern portions of *D. buzzatii*'s distribution range in Argentina. The results of this study based on the analysis of biogeographic trends concur with previous reports: sharp differences among populations living in different geographic areas were detected, but also a substantial fraction of variation in inversion frequencies can be accounted for by altitudinal clines. Moreover, the negative (positive) association of *2st* (*2j*) with altitude is coincident with previous reports based on a reduced dataset (Hasson et al. 1995). Strong associations between the inversion polymorphism, climatic variables, and the trends, again, are coincident with previous information and consistent with what could be expected from the altitudinal clines: the major inversions *2st* and *2j* exhibited direct and inverse clines with temperature, respectively.

However, it should be noted that a multiple regression analysis including both geographic and climatic variables demonstrated that after adjusting for correlated variables the main determinants of variation were climatic variables, particularly mean annual temperature and atmospheric pressure.

The first conclusion of our study, performed more than a decade later of the last studies, emphasizes the stability of the altitudinal and thermal clines reported in Hasson et al. (1995) despite the inclusion of new populations that inhabit ecologically diverse regions. Although clines *per se* cannot be considered as conclusive evidence that population differentiation is a consequence of natural selection shaping the inversion polymorphism, several lines of evidence give strong support to the hypothesis of adaptive differentiation. First, Knibb and Barker (1988) reported significant associations of inversion frequencies with latitude and temperature in Australian populations of *D. buzzatii* that are coincident with the trends observed so far in South America. Second, the strong differentiation observed in inversion frequencies is in sharp contrast with the weak population structure reported for allozymes loci (Rodriguez et al. 2000) and the lack of geographic population structure for sequence variation in mitochondrial DNA (Rossi et al. 1996) and nuclear genes (Gomez and Hasson, 2003; Piccinali et al. 2004, 2007). Finally, it is worth noting that specific inversions (or genes contained within them) affect egg to adult viability, developmental time, and body size related traits (Hasson et al. 1992; Norry et al. 1995; Fanara et al. 1996; Rodríguez et al. 1999; Fernández Iriarte and Hasson 2000). In effect, *2st* and *2j* have negative and positive effects, respectively, on body size and developmental time. Interestingly, size related traits and developmental time vary clinally in *D. buzzatii*, lowland flies tend to be smaller and develop faster than highland (Folguera et al. 2008). Thus, it may be hypothesized that thermal adaptation in *D. buzzatii* may be associated with changes in inversion frequencies; possibly representing a case of hitchhiking whereby frequency changes of specific inversions may reflect the allelic composition (coadapted gene complexes) at specific genes responsive to selection of particular thermal regimes. Thus, inversion polymorphism may be part of the genetic architecture of thermal adaptation via its effects on size related traits and developmental time. Populations adapted to the colder (warmer) regimes in highland (lowland) localities responded by increasing (decreasing) general body size, extending (accelerating) development, and a dramatic reduction in the frequency of *2st* (*2j*).
